# Supplementary material for: From stress to depression: development of extracellular matrix-dependent cognitive impairment following social stress
Source: Sci Rep. 2020 Oct 14;10:17308. doi: 10.1038/s41598-020-73173-2 (PMC7560730; doi:10.1038/s41598-020-73173-2)
Supplement: Supplementary file 1 — Supplementary file1 [file 41598_2020_73173_MOESM1_ESM.pdf]

Supplementary information to:
From stress to depression: Development of extracellular matrix-dependent cognitive impairment following social stress

Maija-Kreetta Koskinen[1], Yvar van Mourik[2], August B. Smit[1], Danai Riga*[,1], Sabine Spijker*[,1]

Contains:

Full method section
Supplemental Figure 1: Prolonged social isolation in adult male Wistar rats does not influence affective and cognitive behavior or hippocampal PNN-counts
Supplemental Figure 2: Stress-induced weight gain across groups and time points
Supplemental Figure 3: Correlation of affective and cognitive phenotypes
Supplemental Figure 4: Whole immunoblot compilation 72 h post-defeat
Supplemental Figure 5: Whole immunoblot compilation week 3 post-defeat
Supplemental Figure 6: Whole gel gelatinase assay 72 h and week 8 post-defeat

Supplemental Table 1: Overview statistics of all main figures.
Supplemental Table 2: Overview data of all main figures.

Full method section

Animals and the social defeat-induced persistent stress paradigm

All experiments were approved by the Vrije University Amsterdam Animal Users Committee and in accordance with the relevant guidelines and regulations. Wistar male rats, 7 weeks old and weighing <200 g upon arrival (Envigo, The Netherlands) were pair-housed and allowed to habituate to the facility for at least 2 weeks before the start of the experiments. Long-Evans male rats (Charles River, UK) were used as residents for this resident-intruder paradigm. The residents were pair-housed with tube-ligated females >1 week before the start of the experiment, in a separate defeat-designated housing room. Females were removed from the resident's cage prior to the defeat sessions. SDPS rats were transferred in the residents' room and underwent 15-minute social defeat sessions daily. During the defeat, the rats were first placed inside the defeat apparatus, while a transparent perforated plexiglass partition wall separated the intruder from the resident, permitting sensory exchange but preventing all physical contact for 5 minutes. The partition wall was then removed and a 5-minute fight phase started. Following the defeat, the rats were separated again and sensory exchange was allowed for another 5-minutes, after which the intruders were placed back to their home cage, and transferred to their housing room. Social defeat was repeated for five consecutive days and each day a new resident was used. From the first defeat session onwards, the SDPS rats were single-housed and kept in social isolation until the end of the experiments. Control rats remained pair-housed throughout the experiment. During defeat days, they were transferred to the residents' room, and let explore an empty social defeat box for the duration of a social defeat session (15 minutes).

Behavioral testing

All animals were habituated to the testing arenas (79 x 57 x 42 cm, plastic) before they underwent any behavioral testing. During the habituation phase, the rats were transported to the video-recording room and let to freely explore an empty testing arena for 10 minutes. The habituation was conducted for 3 times. All behavioral testing was performed during the dark phase under a dim red light.

Social Approach Avoidance – SAA test: The SAA test consisted of 3 phases: habituation, sampling and testing. During habituation, the animals were allowed to explore the empty arena for 5 minutes. This was followed by sampling phase when two empty interaction boxes (23 x 11 x 34 cm, perforated, transparent) were introduced to the arena and the animals were allowed to explore the empty boxes for 5 minutes. After the sampling phase, the rats were placed back to their home-cage, and an unfamiliar Long-Evans rat was placed in one of the boxes. Immediately thereafter, the animals were introduced back to the arena and were allowed to explore the arena again for 3 minutes. The interaction ratio was calculated as time spent near the social target vs. time spent near the empty box: social target zone / (social target zone + empty box zone). Because rats quickly habituate to

novelty, their initial response to the social target is most representative of interest towards (or withdrawal from) social behaviors, thus interaction ratios were calculated based on the first minute of the test.

Object Place Recognition – OPR test: The OPR test consisted of 3 phases: habituation, sampling and testing. During the habituation, the animals were allowed to explore an empty arena for 5 minutes. This was followed by sampling phase upon which two identical objects (8 x 8 x 35 cm, metal, cylinders or cubes) were introduced to the testing arena. After the sampling phase, the rats were placed back to their home-cage. Two objects were replaced with a set of two identical objects but one object was relocated to a different corner within the arena. After a 15-minute interval, the rats were introduced back to the arena and exploration of the objects was analyzed for the first 1 minute. The discrimination index was calculated by measuring the time spent exploring the relocated object compared to the stable object: relocated object / (relocated object + stable object).

Immunohistochemistry

Following transcardial perfusion with and overnight post-fixation in ice-cold 4% PFA in PBS, brains were transferred to 30% sucrose in PBS in 4 °C until sectioned. Free-floating cryostat sections (35 µm) were collected from the dorsal hippocampus (AP -2.40 to -4.56) and stored in PBS+0.02% NaN_3 until further use. Sections were washed 3 times for 10 minutes in PBS, followed by a blocking for 2 h at RT in blocking solution (2.5% BSA, 0.2% Triton, 5% goat serum in PBS). After blocking, the sections were incubated with primary antibodies (mouse anti-chondroitin sulfate proteoglycan 1:1,000, cat-301 MAB5284; rabbit anti-parvalbumin 1:1,000, Swant #235) overnight at 4 °C. This was followed by washing in PBS 4 times 10 minutes at RT, and incubation with fluorescent-conjugated secondary antibodies (anti-mouse-Alexa-488 1:400, Invitrogen A11001; anti-rabbit-Alexa-568 1:400, Invitrogen A11011) for 2 h at RT. Thereafter, the sections were washed 4 times 10 min with PBS at RT. Sections were mounted onto microscope slides using PBS+0.2% gelatin and coverslipped with polyvinyl alcohol mounting medium with DABCO (Merck, 10981).

Images were acquired on a fluorescent microscope (Leica DM5000). The Fiji program, using automated threshold and particle analysis, was used to detect the number of PNN^+ and PV^+ cells and their double immunoreactivity. Specifically, PNNs were identified by applying a “MaxEntropy” threshold, followed by gaussian smoothing. Next, the threshold “Li” was applied and particles sized 100–2,000 and 0–1.00 in circularity were detected as PNNs. On average, PNNs were counted in 2–3 sections per animal, and subsequently averaged per animal. Both n-numbers (section, animal) are given in the legends. PNNs in the pyramidal layer of the CA1 subregion were included in the analysis. To ensure equal conditions between group comparisons, sections from control and SDPS rats per each batch (time-point) were processed simultaneously. False-positive cells were excluded manually during the analysis. During the image acquisition and cell quantification, the researcher was blind to the experimental groups.

Tissue preparation for immunoblotting or zymography

Following decapitation, the brain was removed, and the dorsal hippocampus was immediately dissected on ice[1], and frozen on dry ice.

Immunoblotting – Samples were homogenized in ice-cold 0.32 M sucrose (5% of homogenate was collected as total cell lysate) and then centrifuged at 1000x *g* for 10 minutes. Then, either ECM-bound MMPs for zymography were isolated, or samples were prepared for immunoblotting. For the latter, the supernatant was loaded on top of a sucrose gradient consisting of 0.85 and 1.2 M sucrose. After centrifugation at 100,000x *g* for 2 h, the synaptosome fraction at the interface of 0.85 ⁄ 1.2 M sucrose was collected and then lysed in hypotonic solution.

Protein concentration was determined (Bradford protein assay) and 10 µg of protein was loaded per sample for electrophoresis. The following primary antibodies were used: rabbit anti-aggrecan (1:700, AB1013, Abcam); guinea-pig anti-brevican (1:2,000; generously provided by C.I. Seidenbecher, Magdeburg), mouse anti-neurocan (1:1,000, N0913 Sigma); mouse anti-phosphacan (1:1,000; 3F8, Developmental Studies Hybridoma Bank); mouse anti-versican (1:1,000; 75-324, NeuroMab); mouse anti-Tenascin-R (1:2,000, mTNR-2 Acris Antibodies); rabbit anti-Hapln1 (1:1,000, ab98038 Abcam). After incubation with horseradish peroxidase-conjugated secondary antibody (1:10,000; Dako, Glostrup, Denmark) and visualization with Femto Chemiluminescent Substrate (Thermo Scientific, Rockford, IL, USA), blots were scanned using the Li-Cor Odyssey Fc (Westburg, Leusden, The Netherlands) and analyzed with Image Studio (Li-Cor, Lincoln, NE, USA). Total protein was visualized using trichloro-ethanol staining and scanned using a Gel Doc EZ imager (BioRad, Herculus, CA, USA) and analyzed with Image Lab (BioRad) to correct for differences in sample loading. Normalized data were subsequently log_2-transformed and presented *vs*. those of control samples. In addition, data were compared to the log_2-transformed quantification of perisynaptic ECM protein levels.

MMP extraction & zymography – Tissue was homogenized (50 mM Tris pH 7.4, 10 mM $CaCl_2$ and 0.25% Triton X-100) and centrifuged (6,000x *g*, 30 minutes at 4 °C). The pellet was resuspended (50 mM Tris, pH 7.4, and 0.1 mM $CaCl_2$), heated (60 °C, 15 minutes) and centrifuged (10,000x *g*, 30 minutes at 4 °C). After this, the supernatant was recovered and the protein concentration was determined using Bradford. Thereafter, proteins were precipitated (60% ethanol, 1 minute at 4 °C) and centrifuged (15,000x *g*, 5 minutes at 4 °C). Finally, the pellet was solubilized in non-reducing sample buffer (2% SDS) and heated (37 °C, 15 minutes) before loading on an SDS-PAGE gel containing gelatin (0.1% gelatin, 8% SDS); samples (10 µg of protein) and recombinant mouse MMP-9 as positive control (5 ng, ab39309, Abcam). The gels were washed with 2.5% Triton X-100 (2x 20 minutes) and then incubated for 7 days (50 mM Tris, pH 7.5, 10 mM $CaCl_2$, 1 µM $ZnCl_2$, 1% Triton X-100, and 0.02% sodium azide; 37 °C, 80 rpm). Incubation was followed by Coomassie staining

and destaining (5% HAc) until clear bands were visible. Gels were scanned and analyzed using Gel Doc EZ imager (Biorad, Herculus, CA, USA), and normalized based on Coomassie input.

Statistics

All data were analyzed using IBM SPSS Statistics 24. For group comparisons, two-tailed Student's t-tests (with or without correction for unequal variation) were applied for normally distributed data and Mann-Whitney U-tests otherwise. Data were checked for normality using the Saphiro-Wilk test. All group data are depicted as mean±SEM, with individual data on top. Statistical significance level was set for P-values<0.05, trend for 0.05<P-value<0.10. Details of all statistical testing can be found in Supplementary Table S1, and individual data points can be found in Supplementary Table S2.

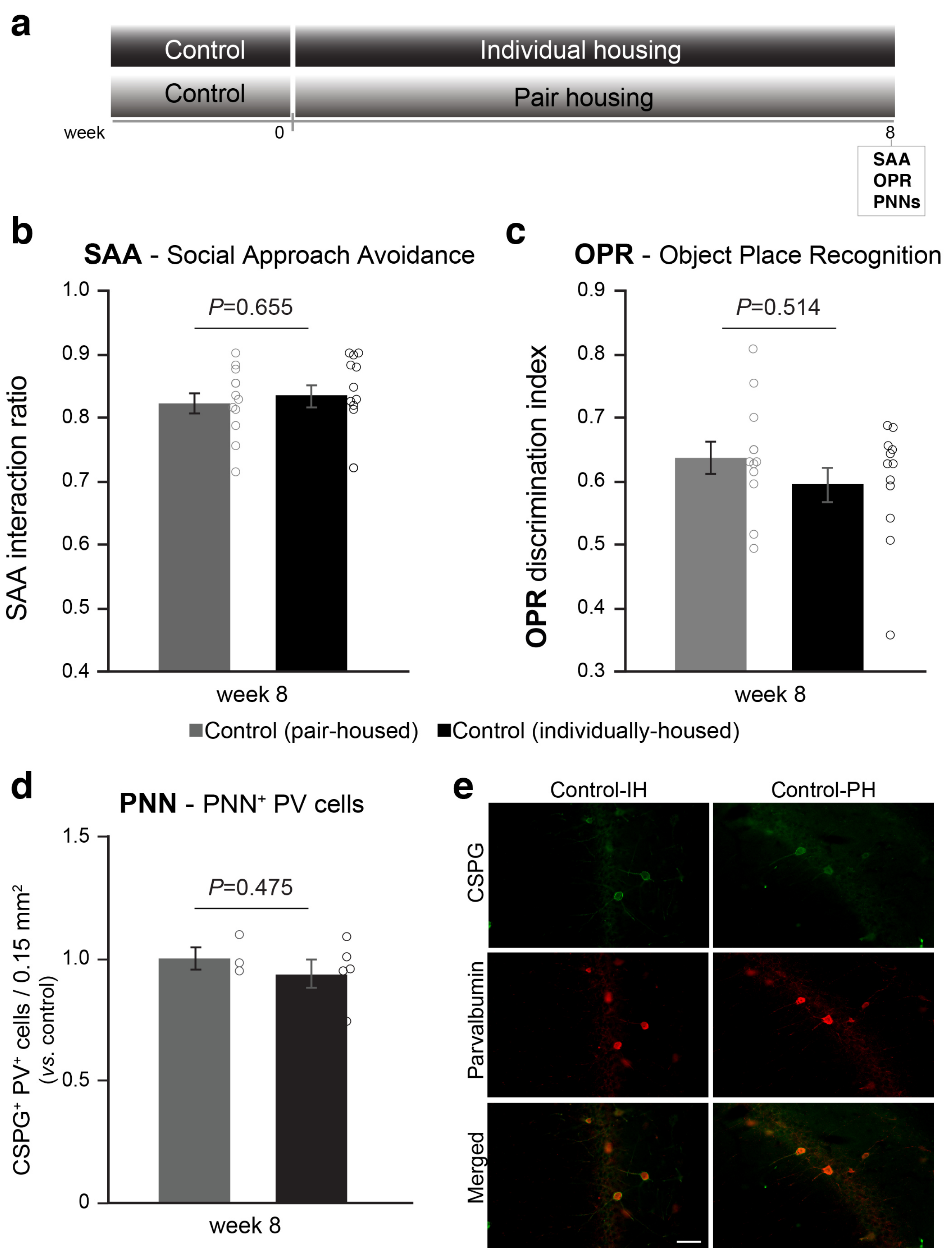

Supplemental Figure 1: **Prolonged social isolation in adult male Wistar rats does not influence affective and cognitive behavior or hippocampal PNN density. a)** Adult rats were exposed to a 5-day sham social defeat paradigm, in which they explored an empty defeat box during 15 minutes. Pair-housed (PH) control rats remained pair-housed throughout the experiment. Individually-housed (IH) control rats experienced a 9-week

period of individual housing starting from the moment of sham defeat onwards. Behavior was assessed with the social approach avoidance (SAA) and object place recognition (OPR) tests, 8 weeks after the last sham social defeat session. The day following the last behavioral test, animals were perfused and processed for PNN-counting. **b,c)** Social isolation had no effect on affective (b, Student's t-test, *P*=0.655), or cognitive (c, Mann-Whitney U test, *P*=0.514) behavior, as shown previously[2]. **d,e)** The number of chondroitin sulfate proteoglycan ($CSPG^+$)-rich perineuronal nets (PNNs, green) onto parvalbumin-expressing (PV^+, red) interneurons were quantified with immunohistochemical (IHC) analysis in the dCA1 (d). No effect of prolonged social isolation was detected (Student's t-test, *P*=0.476), whereas the SDPS paradigm increased PNNs on PV^+ interneurons at this time-point (*cf.* Fig. 2; Riga *et al.*, 2017[2]). Example immunostainings are given; scale bar (75 µm) is indicated (e). Data are expressed as mean±SEM.

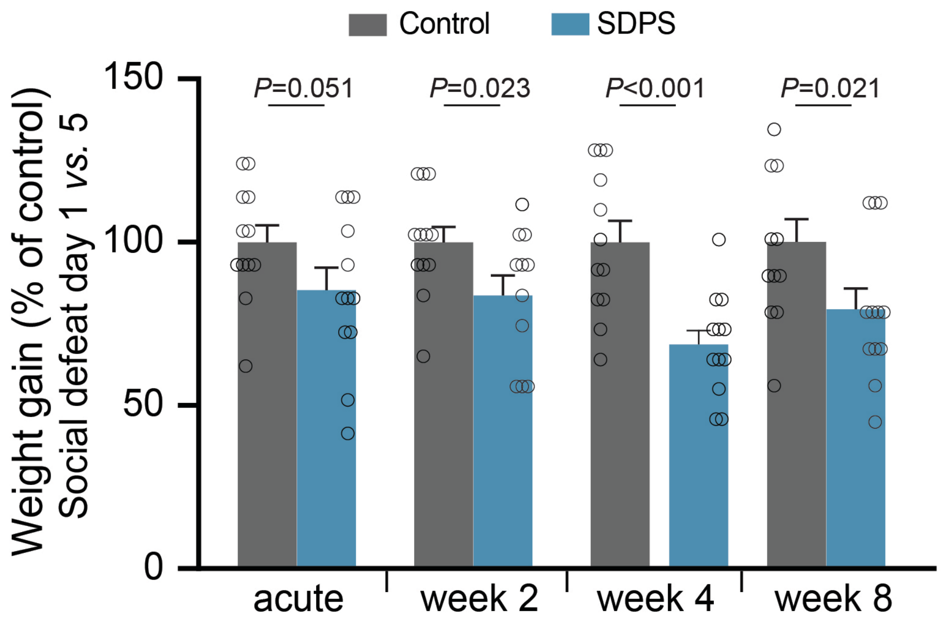

Supplemental Figure 2: Stress-induced weight gain. Weight gain was measured as the difference in weight between social-defeat day 1, prior to defeat, and social-defeat day 5, after defeat, and expressed as % from their corresponding controls in the same batch, to assess similar stress effects of social defeat[2] between batches. A one-way ANOVA (time point/batch) did not reveal a significant effect ($F(3,46)=1.55$; $P=0.216$), indicating that equal stress was experienced across batches. *P*-values (one-tailed Student's t-test) and individual data points are indicated. Data are expressed as mean±SEM.

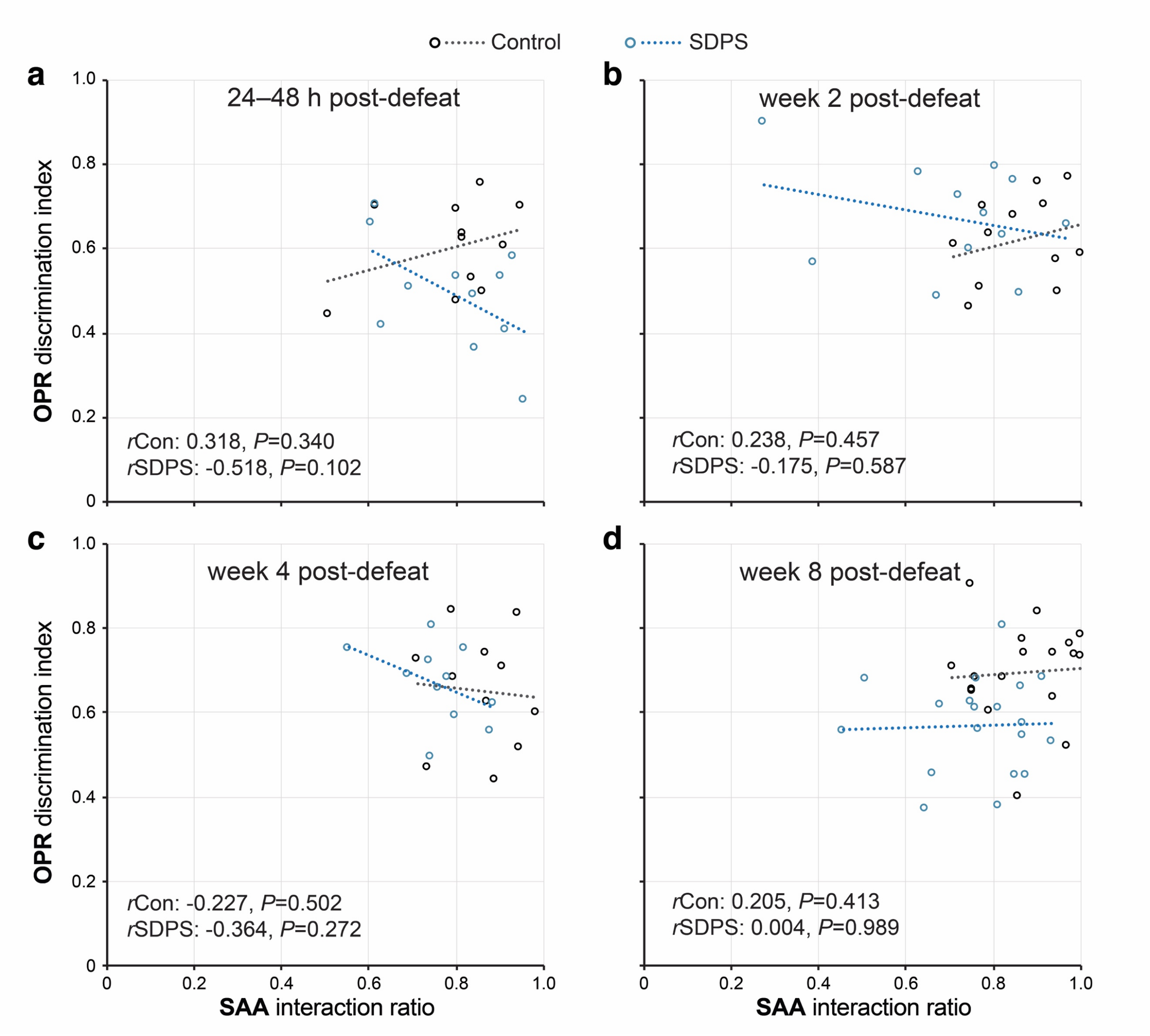

Supplemental Figure 3: Correlation of affective and cognitive phenotypes. SAA interaction ratio and OPR discrimination index were correlated per animal batch undergoing SAA and OPR behavior, either shortly after defeat (**a**), at week 2 (**b**), week 4 (**c**) and week 8 (**d**) post-defeat. Spearman correlations (*r*) and their *P*-values are indicated per group (Con, SDPS), as well as the trendline (dotted line). In addition, Pearson correlation over the entire group per time point indicated the absence of any significant correlations between affective and cognitive parameters ($r_{24\text{-}48\ h}$: -0.146 *P*=0.517; $r_{2\ w}$:-0.212 *P*=0.319; $r_{4\ w}$: -0.223 *P*=0.318; $r_{8\ w}$:0.233 *P*=0.165; see Supplemental Table 1).

Supplementary information
Koskinen et al. – From stress to depression: Development of extracellular matrix-dependent cognitive impairment following social stress

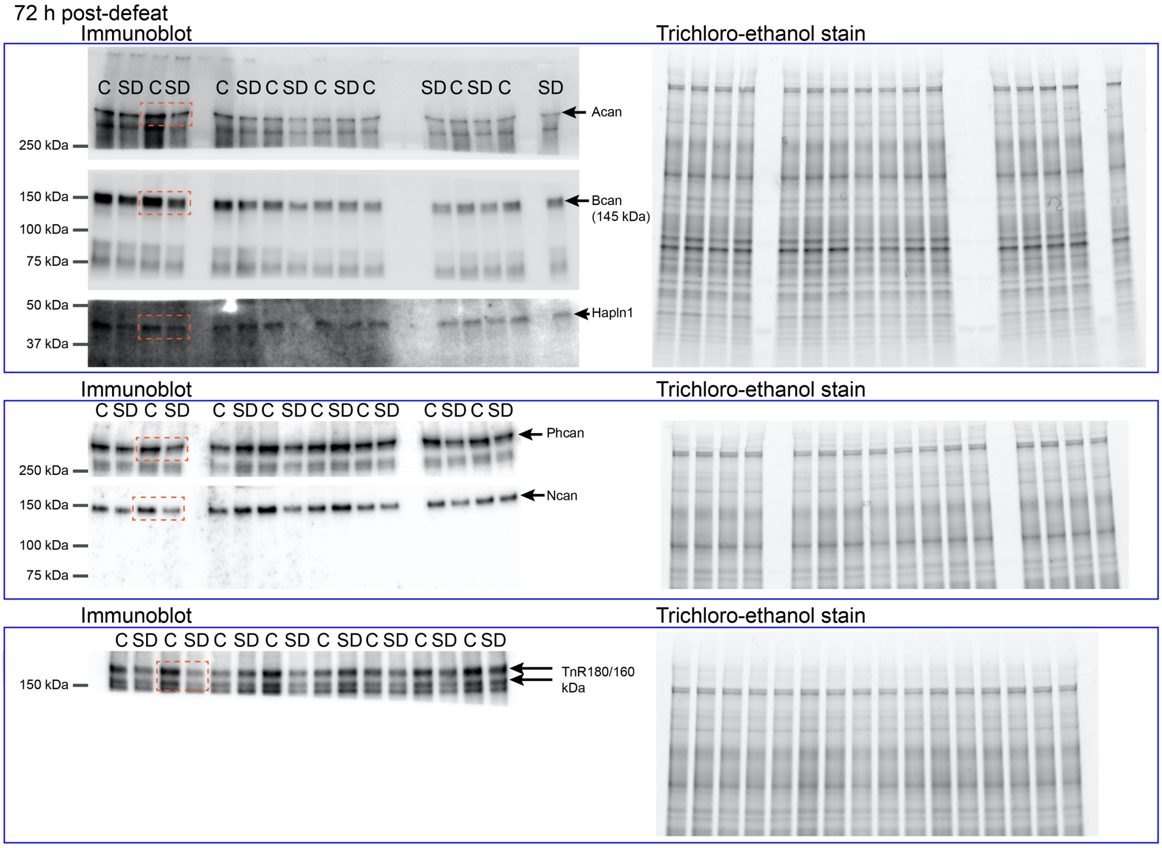

Supplemental Figure 4. Whole immunoblot compilation 72 h post defeat. Whole immunoblots (left) and trichloro-ethanol stained gels (right) are shown from which sections (orange dashed rectangle) are included in Figure 3 for the 72 h post-defeat time-point (C=control animal; SD=SDPS animal). Molecular weights are indicated.

Supplementary information
Koskinen et al. – From stress to depression: Development of extracellular matrix-dependent cognitive impairment following social stress

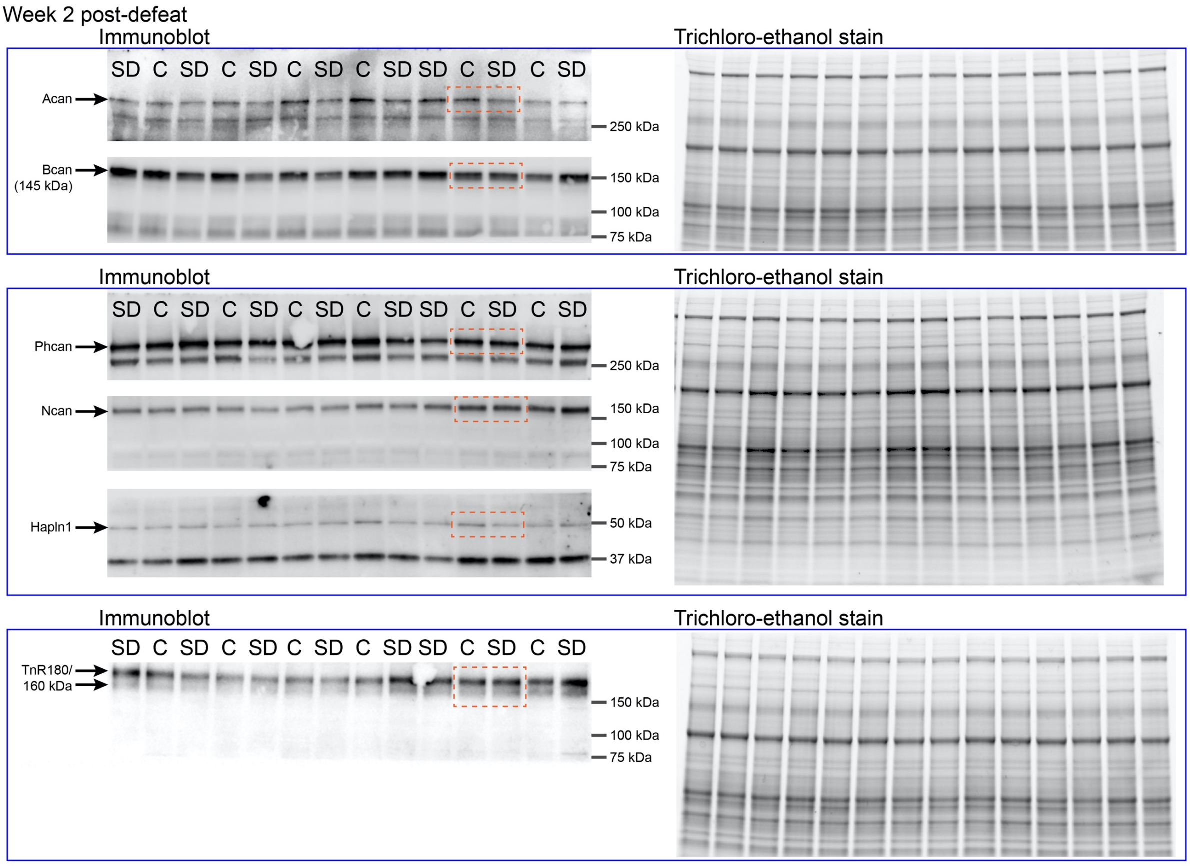

Supplemental Figure 5. Whole immunoblot compilation week 2 post defeat. Whole immunoblots (left) and trichloro-ethanol stained gels (right) are shown from which sections (orange dashed rectangle) are included in Figure 3 for the week 2 post-defeat time-point (C=control animal; SD=SDPS animal). Molecular weights are indicated.

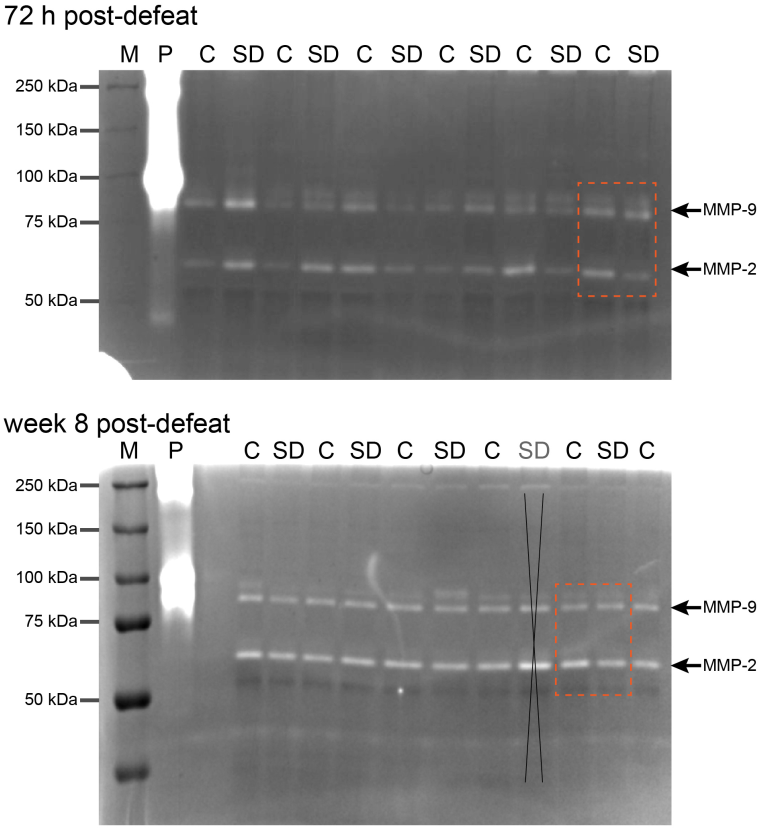

Supplemental Figure 6. Whole gel gelatinase assay 72 h and week 8 post-defeat. Whole gels for in gel zymography (72 h, up; week 8, down) are shown from which sections (orange dashed rectangle) are included in Figure 4. The mature form of MMP-2 and MMP-9 (white bands), as well as molecular weights are indicated. Sample normalization for loading differences was performed on the Coomassie-stained bands. M, marker; P, positive control of human MMP-9, C=control animal; SD=SDPS animal. Note that 1 SDPS sample (crossed, week 8) was considered an outlier at the protein level and for MMP activity.

Supplementary information
Koskinen et al. – From stress to depression: Development of extracellular matrix-dependent cognitive impairment following social stress

Supplemental Table 1. Overview statistics all figures. Shown are the results and details of the 2-tailed statistical analyses (type of test, n-number, t-value, Df, *P*-value) for the data shown in Figure 1 (behavior), Supplemental Figure 1 (isolation on behavior), Supplemental Figure 2 (weight gain), Supplemental Figure 3 (correlation of behavior), Figure 2 (PNN staining), Figure 3 (Immunoblot), and Figure 4 (MMP gelatinase activity) in SDPS *vs.* control (CON) animals for the indicated time points. Significance (*P*<0.050) is indicated in bold, trend (*P*<0.100) in underlined; tests with unequal variance are in italics.

Figure	***Statistical test*** *(n_{Con}, n_{SDPS})*	***Statistics*** *(t-value, Df)*	***P-value***
1b	**Mann-Whitney U test**		
	CON vs. SDPS 24 h (11, 11)		*P* = 0.935
	CON *vs.* SDPS week 8 (18, 19)		***P* = 0.026**
	**Unpaired t-test**		
	CON *vs.* SDPS week 2 (12, 12)	t(22) = 2.352	***P* = 0.028**
	CON *vs.* SDPS week 4 (11, 11)	t(20) = 2.456	***P* = 0.023**
	CON *vs.* fictive 24 h	(t20) = 5.484	**$P < 10^{-4}$**
	CON *vs.* fictive week 2	t(22) = 8.888	**$P < 10^{-4}$**
	CON *vs.* fictive week 4	t(20) = 9.457	**$P = 10^{-4}$**
	CON *vs.* fictive week 8	t(34) = 11.104	**$P < 10^{-4}$**
	SDPS *vs.* fictive 24 h	t(20) = 5.162	**$P < 10^{-4}$**
	SDPS *vs.* fictive week 2	t(22) = 2.575	***P* = 0.017**
	SDPS *vs.* fictive week 4	t(20) = 6.733	**$P < 10^{-4}$**
	SDPS *vs.* fictive week 8	t(36) = 6.382	**$P < 10^{-4}$**
1c	**Unpaired t-test**		
	CON *vs.* SDPS 48 h (11, 11)	t(20) = 2.189	***P* = 0.041**
	CON *vs.* SDPS week 2 (12, 12)	t(22) = -1.067	*P* = 0.298
	CON *vs.* SDPS week 4 (11, 11)	t(20) = -0.271	*P* = 0.789
	CON *vs.* SDPS week 8 (18, 19)	t(35) = 3.366	***P* = 0.002**
	CON *vs.* fictive 48 h	t(20) = 2.349	***P* = 0.029**
	CON *vs.* fictive week 2	t(22) = 2.944	***P* = 0.008**
	CON *vs.* fictive week 4	t(20) = 2.583	***P* = 0.018**
	CON *vs.* fictive week 8	t(34) = 5.094	***P* < 0.001**
	SDPS *vs.* fictive 48 h	t(20) = -0.116	*P* = 0.908
	SDPS *vs.* fictive week 2	t(22) = 3.356	***P* = 0.003**
	SDPS *vs.* fictive week 4	t(20) = 4.106	***P* = 0.001**
	SDPS *vs.* fictive week 8	t(36) = 1.890	<u>*P* = 0.067</u>
S1b	**Unpaired t-test (SAA)**		
	CONpair *vs.* CONiso week 8 (12, 12)	t(22) = -0.439	*P* = 0.665
	**Mann-Whitney U test (OPR)**		
	CONpair vs. CONiso week 8 (11, 11)		*P* = 0.514
	CONiso *vs.* fictive week 8		***P* = 0.001**
	**Unpaired t-test (OPR)**		
	CONpair *vs.* fictive week 8	t(22) = 3.798	***P* = 0.001**
S1c	**Unpaired t-test (PNN)**		
	CONpair *vs.* CONiso week 8 (3, 5)	t(6) = 0.761	*P* = 0.476
S2	**One way ANOVA**		
	Weight gain; control-normalized values (per time point) of SDPS batches / time points	F(3,46) = 1.55	*P* = 0.216
S3a–d	**Pearson correlation SAA & OPR per time point**		
	$SAA_{24\,h}$ *vs* $OPR_{48\,h}$ (22)	$r_{24\text{-}48\,h}$ = -0.146	*P* = 0.517
	$SAA_{2\,w}$ *vs* OPR_{2w} (24)	$r_{2\,w}$ = -0.212	*P* = 0.319
	$SAA_{4\,w}$ *vs* $OPR_{4\,w}$ (22)	$r_{4\,w}$ = -0.223	*P* = 0.318
	$SAA_{8\,w}$ *vs* $OPR_{8\,w}$ (37)	$r_{8\,w}$ = 0.233	*P* = 0.165
	**Spearman correlation SAA & OPR per group per time point**		
	**Control**		
	$SAA_{24\,h}$ *vs* $OPR_{48\,h}$ (11)	$r_{24\text{-}48\,h}$ = 0.318	*P* = 0.340
	$SAA_{2\,w}$ *vs* OPR_{2w} (12)	$r_{2\,w}$ = 0.238	*P* = 0.457
	$SAA_{4\,w}$ *vs* $OPR_{4\,w}$ (11)	$r_{4\,w}$ = -0.227	*P* = 0.502
	$SAA_{8\,w}$ *vs* $OPR_{8\,w}$ (18)	$r_{8\,w}$ = 0.205	*P* = 0.413

	**SDPS**<br>$SAA_{24\ h}$ *vs* $OPR_{48\ h}$ (11)<br>$SAA_{2\ w}$ *vs* OPR_{2w} (12)<br>$SAA_{4\ w}$ *vs* $OPR_{4\ w}$ (11)<br>$SAA_{8\ w}$ *vs* $OPR_{8\ w}$ (19)	 $r_{24\text{-}48\ h}$ = -0.518<br>$r_{2\ w}$ = -0.175<br>$r_{4\ w}$ = -0.364<br>$r_{8\ w}$ = 0.004	 *P* = 0.102<br>*P* = 0.587<br>*P* = 0.272<br>*P* = 0.989
2b	**Unpaired t-test**<br>CON *vs*. SDPS 72 h (5, 4)<br>CON *vs*. SDPS week 2 (4, 3)<br>CON *vs*. SDPS week 4 (3, 4)<br>CON *vs*. SDPS week 8 (4, 4)	 t(7) = 2.489<br>t(5) = -1.730<br>t(5) = -1.833<br>t(6) = -4.966	 ***P* = 0.042**<br>*P* = 0.144<br>*P* = 0.126<br>***P* = 0.001**
2d	**Unpaired t-test**<br>CON *vs*. SDPS 72h<br>CON *vs*. SDPS week 2<br>CON *vs*. SDPS week 4<br>CON *vs*. SDPS week 8	 t(5) = -0.030<br>t(5) = -1.673<br>t(5) = -1.940<br>*t(3.850) = -1.105*	 *P* = 0.977<br>*P* = 0.155<br>*P* = 0.110<br>*P = 0.333*
3b,c	*Log_2 transformed data*<br>**72 h**<br>**Unpaired t-test**<br>CON *vs*. SDPS Acan (8, 7)<br>CON *vs*. SDPS Bcan (8, 7)<br>CON *vs*. SDPS Ncan (8, 7)<br>CON *vs*. SDPS Pcan (8, 7)<br>CON *vs*. SDPS TenR160 (8, 6)<br>CON *vs*. SDPS TenR180 (8, 7)<br><br>**Mann-Whitney U test**<br>CON *vs*. SDPS Hapln1 (8, 7)<br><br>**Week 2**<br>**Unpaired t-test**<br>CON *vs*. SDPS Acan (6, 8)<br>CON *vs*. SDPS Bcan (6, 8)<br>CON *vs*. SDPS Ncan (6, 8)<br>CON *vs*. SDPS Pcan (6, 8)<br>CON *vs*. SDPS TenR160 (6, 8)<br>CON *vs*. SDPS TenR180 (6, 8)<br>CON *vs*. SDPS Hapln1<br><br>**Week 8***<br>**Unpaired t-test**<br>CON *vs*. SDPS Acan (4, 5)<br>CON *vs*. SDPS Bcan (4, 5)<br>CON *vs*. SDPS Pcan (4, 5)<br>CON *vs*. SDPS TenR160 (4, 4)<br>CON *vs*. SDPS TenR180 (4, 4)<br>CON *vs*. SDPS Hapln1<br><br>**Mann-Whitney U test**<br>CON *vs*. SDPS Ncan (4, 5)	 <br><br>t(13) = 0.779<br>t(13) = 2.300<br>t(13) = 2.453<br>t(13) = 2.610<br>t(13) = 3.213<br>t(13) = 2.197<br><br><br><br><br><br><br>t(12) = 1.083<br>t(12) = 0.009<br>t(12) = -0.312<br>t(12) = 0.435<br>t(12) = -1.021<br>t(12) = -0.495<br>t(12) = 0.710<br><br><br><br>t(7) = -1.107<br>t(7) = -3.582<br>t(7) = -3.563<br>t(7) = -1.999<br>t(7) = -1.505<br>t(7) = -3.226	 <br><br>*P* = 0.450<br>***P* = 0.039**<br>***P* = 0.029**<br>***P* = 0.022**<br>***P* = 0.007**<br>***P* = 0.047**<br><br><br>***P* = 0.021**<br><br><br><br>*P* = 0.300<br>*P* = 0.993<br>*P* = 0.760<br>*P* = 0.671<br>*P* = 0.327<br>*P* = 0.629<br>*P* = 0.491<br><br><br><br>*P* = 0.305<br>***P* = 0.009**<br>***P* = 0.009**<br>*P* = 0.093<br>*P* = 0.183<br>***P* = 0.015**<br><br><br>***P* = 0.016**
4b,c	**72 h**<br>**Unpaired t-test**<br>CON *vs*. SDPS MMP-2 (6, 6)<br>CON *vs*. SDPS MMP-9 (6, 6)<br><br>**Week 8**<br>**Unpaired t-test**<br>CON *vs*. SDPS MMP-2 (6, 4)<br>CON *vs*. SDPS MMP-9 (6, 4)	 <br>t(10) = -0.513<br>t(10) = -1.09<br><br><br><br>t(8) = 2.398<br>t(8) = 2.272	 <br>*P* = 0.619<br>*P* = 0.301<br><br><br><br>***P* = 0.043**<br>*P* = 0.239

* Note, the week 8 immunoblot data (non-log2-transformed) have been published before[2], and are not repeated to adhere to the 3R-principle of animal research.

Supplementary information

Koskinen et al. – From stress to depression: Development of extracellular matrix-dependent cognitive impairment following social stress

Supplemental Table 2. Overview data all main figures. Shown are all individual data points of Figures 1–4

SAA behavior Figure 1

CON	24 h	>2 w	>4 w	>8 w
C1	0.507592	0.916828	0.985011	0.792079
C2	0.816068	0.949710	0.711779	0.987730
C3	0.860465	0.946237	0.943277	0.937500
C4	0.856818	0.792517	0.794372	n/a
C5	0.836066	0.777320	0.870712	0.869565
C6	0.950216	0.904523	n/a	0.976431
C7	0.802139	0.974052	0.888889	0.706215
C8	0.801887	1.000000	0.735602	0.747748
C9	0.617978	0.846482	0.906810	0.824324
C10	0.911308	0.768737	0.940803	0.751634
C11	0.815789	0.710345	0.866906	0.753659
C12	n/a	0.744792	0.791155	0.939597
C13				**1.000000**
C14				0.969793
C15				0.856164
C16				0.759664
C17				0.873282
C18				0.903010
C19				**1.000000**

Fictive data belonging to each data set (average 0.50 with equal variance as correspondin

	24 h	*>2 w*	*>4 w*	*>8 w*
C1	*0.209744*	*0.555866*	*0.627255*	*0.422724*
C2	*0.518220*	*0.588748*	*0.354023*	*0.618375*
C3	*0.562617*	*0.585275*	*0.585521*	*0.568145*
C4	*0.558970*	*0.431555*	*0.436616*	
C5	*0.538218*	*0.416358*	*0.512956*	*0.500210*
C6	*0.652369*	*0.543561*		*0.607076*
C7	*0.504291*	*0.613090*	*0.531133*	*0.336859*
C8	*0.504039*	*0.639038*	*0.377846*	*0.378392*
C9	*0.320130*	*0.485520*	*0.549054*	*0.454969*
C10	*0.613460*	*0.407775*	*0.583047*	*0.382279*
C11	*0.517942*	*0.349383*	*0.509150*	*0.384303*
C12		*0.383830*	*0.433399*	*0.570242*
C13				*0.630645*
C14				*0.600438*
C15				*0.486809*
C16				*0.390309*
C17				*0.503927*
C18				*0.533655*
C19				*0.630645*

SDPS	24 h	>2 w	>4 w	>8 w
SD1	0.801636	0.969121	0.879493	0.864583
SD2	0.902637	0.846154	0.817778	0.643799
SD3	0.956000	0.722567	0.746392	0.763298
SD4	n/a	0.387978	0.740099	0.935574
SD5	0.914634	0.746324	n/a	0.760291
SD6	0.617978	0.781377	0.689008	0.869281
SD7	0.606061	0.821138	0.781443	0.874564
SD8	0.930830	0.860465	0.759300	0.913934
SD9	0.841509	0.670968	0.799578	0.507937
SD10	0.842995	0.629310	0.553444	0.678670
SD11	0.630137	0.274892	0.742931	0.810734
SD12	0.694954	0.805263	0.886463	0.456576
SD13				0.767442
SD14				0.660819
SD15				0.868526
SD16				0.823276
SD17				0.813299
SD18				0.750000
SD19				0.851190

	24 h	*>2 w*	*>4 w*	*>8 w*
SD1	*0.507148*	*0.759491*	*0.616226*	*0.595436*
SD2	*0.608149*	*0.636524*	*0.554512*	*0.374652*
SD3	*0.661512*	*0.512938*	*0.483126*	*0.494151*
SD4		*0.178348*	*0.476833*	*0.666427*
SD5	*0.620146*	*0.536694*		*0.491143*
SD6	*0.323489*	*0.571747*	*0.425742*	*0.600134*
SD7	*0.311572*	*0.611508*	*0.518177*	*0.605417*
SD8	*0.636342*	*0.650835*	*0.496034*	*0.644787*
SD9	*0.547021*	*0.461338*	*0.536312*	*0.238789*
SD10	*0.548507*	*0.419681*	*0.290178*	*0.409523*
SD11	*0.335649*	*0.065262*	*0.479664*	*0.541587*
SD12	*0.400466*	*0.595633*	*0.623197*	*0.187429*
SD13				*0.498295*
SD14				*0.391672*
SD15				*0.599379*
SD16				*0.554129*
SD17				*0.544152*
SD18				*0.480853*
SD19				*0.582043*

Preference to the social target: time spent in social target zone / (social target zone + empty box zone).

OPR behaviour

Fictive data belonging to each data set (average 0.50 with equal variance as corresponding data set)

CON	48 h	>2 w	>4 w	>8 w	*48 h*	*>2 w*	*>4 w*	*>8 w*
C1	0.440816	0.701149	0.598456	0.601036	*0.335842*	*0.578954*	*0.447409*	*0.405919*
C2	0.622283	0.496296	0.726471	0.736434	*0.517308*	*0.374101*	*0.575424*	*0.541317*
C3	0.497890	0.571429	0.513453	0.632432	*0.392916*	*0.449233*	*0.362406*	*0.437315*
C4	0.754875	0.632850	0.681564	n/a	*0.649900*	*0.510655*	*0.530517*	
C5	0.530120	0.700337	0.621818	0.771318	*0.425146*	*0.578141*	*0.470771*	*0.576201*
C6	0.700831	0.757256	n/a	0.759494	*0.595856*	*0.635061*		*0.564377*
C7	0.691589	0.766990	0.436725	0.705714	*0.586614*	*0.644795*	*0.285678*	*0.510597*
C8	0.476309	0.585470	0.466867	0.900794	*0.371335*	*0.463275*	*0.315821*	*0.705677*
C9	0.699647	0.676056	0.705882	0.679537	*0.594672*	*0.553861*	*0.554835*	*0.484420*
C10	0.606557	0.506812	0.832765	0.646875	*0.501583*	*0.384617*	*0.681718*	*0.451758*
C11	0.633803	0.610429	0.738506	0.650558	*0.528828*	*0.488234*	*0.587459*	*0.455440*
C12	n/a	0.461268	0.839009	0.739645		*0.339072*	*0.687962*	*0.544528*
				0.730769				*0.535652*
				0.516779				*0.321661*
				0.398281				*0.203164*
				0.682657				*0.487540*
				0.740625				*0.545508*
				0.836735				*0.641618*
				0.782427				*0.587310*

SDPS	48 h	>2 w	>4 w	>8 w	*48 h*	*>2 w*	*>4 w*	*>8 w*
SD1	0.530892	0.654434	0.554252	0.659524	*0.537473*	*0.482523*	*0.389606*	*0.590397*
SD2	0.531977	0.759669	0.751656	0.370370	*0.538558*	*0.587757*	*0.587009*	*0.301244*
SD3	0.240166	0.724590	0.804878	0.678295	*0.246746*	*0.552679*	*0.640232*	*0.609168*
SD4	n/a	0.565217	0.721951	0.528926		*0.393306*	*0.557305*	*0.459799*
SD5	0.404922	0.599455	n/a	0.609489	*0.411502*	*0.427543*		*0.540362*
SD6	0.701299	0.679389	0.688385	0.572193	*0.707879*	*0.507478*	*0.523739*	*0.503066*
SD7	0.657692	0.631429	0.682493	0.450581	*0.664273*	*0.459517*	*0.517846*	*0.381455*
SD8	0.581140	0.492308	0.657005	0.679487	*0.587721*	*0.320396*	*0.492359*	*0.610360*
SD9	0.490625	0.487085	0.588921	0.678125	*0.497206*	*0.315173*	*0.424275*	*0.608998*
SD10	0.363636	0.777293	0.750636	0.615063	*0.370217*	*0.605381*	*0.585990*	*0.545936*
SD11	0.416490	0.900262	0.492918	0.377990	*0.423071*	*0.728351*	*0.328272*	*0.308864*
SD12	0.508772	0.791809	0.618012	0.552743	*0.515353*	*0.619897*	*0.453366*	*0.483616*
				0.557377				*0.488250*
				0.452229				*0.383102*
				0.544747				*0.475620*
				0.805556				*0.736429*
				0.607029				*0.537902*

Supplementary information

Koskinen et al. – From stress to depression: Development of extracellular matrix-dependent cognitive impairment following social stress

			0.623377				*0.554250*
			0.450311				*0.381184*

Preference to the relocated object: time spent on relocated object/ (relocated object + stable object)

Note that separate batches of animals were used, i.e. animal C1 @ SAA 24 h is different from animal C1 @ SAA 4 weeks.

However, each animal underwent first the SAA task and then the OPR task.

PNN/PV number Figure 2

**CON**	72 h	>2 w	>4 w	>8 w
C1	5.00	5.21	5.00	3.64
C2	4.71	5.00	4.79	3.89
C3	5.33	4.83	n/a	3.55
C4	n/a	5.32	4.67	4.22
C5	5.31			
C6	5.22			

**SDPS**	72 h	>2 w	>4 w	>8 w
SD1	3.79	5.61	6.27	4.61
SD2	5.62	5.10	5.23	4.88
SD3	3.95	4.89	4.92	4.03
SD4	3.63	4.69	5.07	4.73
SD5	3.83			
SD6	4.44			

Total PV number

**CON**	72 h	>2 w	>4 w	>8 w
C1	6.06	5.96	5.94	5.08
C2	5.36	5.73	5.36	5.18
C3	6.33	5.31	n/a	3.89
C4	n/a	5.61	5.58	4.11

**SDPS**	72 h	>2 w	>4 w	>8 w
SD1	6.75	6.17	7.45	5.24
SD2	6.28	6.05	6.00	4.95
SD3	4.58	5.72	6.08	5.00
SD4	6.13	n/a	6.27	4.63

PNN/PV numbers normalised to Control

**CON**	72 h	>2 w	>4 w	>8 w
C1	0.977	1.023	1.038	0.951
C2	0.922	0.982	0.993	1.017
C3	1.043	0.948	n/a	0.929
C4	n/a	1.046	0.969	1.104
C5	1.038			
C6	1.021			

**SDPS**	72 h	>2 w	>4 w	>8 w
SD1	0.741	1.102	1.302	1.206
SD2	1.099	1.002	1.086	1.276
SD3	0.772	0.961	1.022	1.054
SD4	0.709	0.921	1.052	1.237
SD5	0.749			
SD6	0.867			

Total PV number normalised to control

**CON**	72 h	>2 w	>4 w	>8 w
C1	1.024	1.054	1.057	1.113
C2	0.906	1.014	0.952	1.135
C3	1.071	0.939	n/a	0.852
C4	n/a	0.993	0.991	0.900
C5				

**SDPS**	72 h	>2 w	>4 w	>8 w
SD1	1.141	1.092	1.325	1.148
SD2	1.061	1.070	1.067	1.084
SD3	0.774	1.013	1.080	1.095
SD4	1.035	n/a	1.114	1.014
SD5				

Note that separate batches of animals were used, i.e. animal C1 @ 72 h is different from animal C1 @ 4 weeks.

Figure 3 Immunoblot data

Supplementary information

Koskinen et al. – From stress to depression: Development of extracellular matrix-dependent cognitive impairment following social stress

72 h	Acan	Bcan	Ncan	Phcan	TnR160	TnR180	Hapln
Con1	0.57	-0.09	-0.28	-0.28	-0.32	-0.33	0.54
Con2	1.03	-0.23	0.15	0.04	0.09	0.37	0.24
Con3	0.13	-0.30	-0.09	-0.59	0.05	-0.49	-1.11
Con4	-0.32	-0.17	0.62	0.50	0.26	0.34	0.17
Con5	-0.45	0.09	0.17	0.26	0.23	-0.02	-0.03
Con6	-0.65	0.27	-0.36	-0.16	-0.44	-0.47	-0.19
Con7	-0.67	-0.05	-0.22	0.01	0.12	0.03	-0.37
Con8	-0.79	0.35	-0.26	-0.05	-0.14	0.26	0.19
SDPS1	0.39	-0.41	-0.81	-0.87	-0.48	-0.77	-1.19
SDPS2	0.15	-0.59	-0.74	-0.84	-0.60	-0.99	-1.08
SDPS3	-0.38	-0.67	-0.13	-0.10	-0.63	-0.47	-0.79
SDPS4	-0.70	-0.50	-0.14	-0.24	-0.10	-0.25	-3.85
SDPS5	-0.67	0.03	-0.38	-0.06	-0.48	-0.43	-0.09
SDPS6	-0.84	-0.35	-0.62	-0.80	n/a	-0.40	-0.55
SDPS7	-0.60	0.13	-0.19	-0.57	-0.27	0.16	-0.06

>2 weeks	Acan	Bcan	Ncan	Phcan	TnR160	TnR180	Hapln
Con1	-0.8217	-0.4179	0.2605	-0.2013	0.2941	0.1237	0.2586
Con2	0.2052	-0.0129	0.2045	-0.2422	0.2938	0.2631	0.2013
Con3	0.8086	0.2110	-0.1703	0.1745	0.0401	0.1834	-0.1520
Con4	-0.0729	-0.1628	-0.2225	-0.1056	-0.2322	-0.3345	-0.0971
Con5	-0.1778	0.0054	-0.1152	0.2848	-0.5370	-0.5969	-0.0432
Con6	-0.5249	0.2701	-0.0279	0.0123	-0.0301	0.1645	-0.2371
SDPS1	-0.3082	0.1391	0.5801	0.0158	0.9024	0.6210	0.0994
SDPS2	-0.5462	-0.0741	0.3830	-0.1427	0.5417	0.4515	0.1837
SDPS3	0.4718	0.2545	0.1199	0.0287	0.5203	0.2847	-0.4204
SDPS4	0.1648	0.0377	-0.0198	0.0426	0.5992	0.3013	0.0319
SDPS5	-0.1909	-0.2402	-0.4868	-0.3308	-0.2551	-0.5001	-0.5204
SDPS6	-2.3080	-0.5759	-0.3352	-0.4125	-0.5465	-0.5987	0.1374
SDPS7	-0.4972	-0.1126	-0.1675	-0.0353	-0.2430	-0.2584	-0.0050
SDPS8	-1.0658	0.4178	0.2445	0.3138	0.2028	0.3321	-0.3669

>8 weeks	Acan	Bcan	Ncan	Phcan	TnR160	TnR180	Hapln
Con1	0.67131	0.45242	-0.00937	0.15845	-0.28810	-0.39067	0.51954
Con2	-0.21631	-0.40992	0.10025	-0.16889	0.06759	0.26508	-0.14746
Con3	-0.76507	-0.31397	-0.34660	-0.33604	-0.12660	-0.27155	-0.29478
Con4	-0.06147	0.10377	0.19922	0.26564	0.28341	0.27154	-0.23708
SDPS1	1.31676	1.20241	1.18922	1.33367	0.22808	0.12979	1.17424
SDPS2	0.93178	1.41809	1.18738	1.18628	0.15438	-0.08361	1.12194
SDPS3	0.37677	1.16455	1.12848	0.69289	0.93895	0.74884	0.27348
SDPS4	-0.61733	0.29471	1.03171	0.75610	n/a	n/a	1.10373
SDPS5	0.08146	0.79547	0.35134	0.28869	1.75111	1.67605	0.48498

Note, data have been normalized to total protein input and subsequently log2-transformed.
Note, the 9 week data have been published before (non-log2-transformed): Riga et al Science Translational Medicine 2017

Supplementary information

Koskinen et al. – From stress to depression: Development of extracellular matrix-dependent cognitive impairment following social stress

Note that separate batches of animals were used, i.e. animal C1 @ 72 h is different from animal C1 @ 4 weeks.

Figure 4 Zymography

MMP-2

**CON**	72 h	>8 w
C1	0.60	1.15
C2	0.50	0.79
C3	1.23	1.02
C4	0.44	1.09
C5	1.94	1.19
C6	1.29	0.75

**SDPS**	72 h	>8 w
SD1	2.12	0.68
SD2	1.84	0.85
SD3	0.76	0.82
SD4	1.04	0.70
SD5	0.63	
SD6	0.70	

MMP-9

**CON**	72 h	>8 w
C1	1.10	1.13
C2	0.67	1.02
C3	1.34	1.23
C4	0.47	0.95
C5	0.99	0.66
C6	1.43	1.01

**SDPS**	72 h	>8 w
SD1	2.71	0.66
SD2	1.05	1.06
SD3	0.50	0.92
SD4	1.41	0.73
SD5	0.93	
SD6	1.68	

Note that separate batches of animals were used, i.e. animal C1 @ 72 h is different from animal C1 @ 8 weeks.

Supplementary information
Koskinen et al. – From stress to depression: Development of extracellular matrix-dependent cognitive impairment following social stress

References

1. Spijker, S. Dissection of Rodent Brain Regions. In *Neuromethods* **57**, pp 13-26, Totowa, NJ: Humana press (2011).
2. Riga, D. *et al.* Hippocampal extracellular matrix alterations contribute to cognitive impairment associated with a chronic depressive-like state in rats. *Sci Transl Med* **9**, eaai8753 (2017).
